# Embedded motivational interviewing combined with a smartphone application to increase physical activity in people with sub-acute low back pain: a cluster randomised controlled trial

**DOI:** 10.1016/j.bjpt.2024.101091

**Published:** 2024-06-16

**Authors:** Jason Holden, Paul O'Halloran, Megan Davidson, Jeff Breckon, Wenny Rahayu, Melissa Monfries, Nicholas F. Taylor

**Affiliations:** a La Trobe University, School of Allied Health, Bundoora, Victoria, Australia; b La Trobe University, School of Psychology and Public Health, Bundoora, Victoria, Australia; c Academy of Sport and Physical Activity, Sheffield Hallam University, Sheffield, United Kingdom; d La Trobe University, Office of Engineering and Mathematical Sciences, Bundoora, Victoria, Australia; e La Trobe University, School of Allied Health, Human Services and Sport, Bundoora, Victoria, Australia; f Eastern Health, Arnold St, Box Hill, Victoria, Australia

**Keywords:** Low back pain, Motivational interviewing, Randomised controlled trial, Smartphone application, app

## Abstract

**Background:**

Moderately vigorous physical activity (PA) may be beneficial for people with sub-acute low back pain (LBP), but may initially be painful for patients and challenging for physical therapists to facilitate.

**Objectives:**

This study investigated motivational interviewing (MI) delivered by physical therapists and a smartphone app for increasing PA in people with LBP.

**Methods:**

A mixed methods cluster randomised controlled trial involving 46 adults with LBP in Melbourne, Australia. Participants attended weekly 30-min physical therapy consultations for 6 weeks. Experimental group physical therapists were taught to embed MI into consultations and patients were provided with a self-directed app. The primary outcome was accelerometer-derived moderately vigorous PA. Secondary outcomes were LBP disability (Oswestry Disability Index), functional capacity (Patient Specific Functional Scale), and self-efficacy (Pain Self-Efficacy Questionnaire). Between-group differences were analysed by ANCOVA post-intervention.

**Results:**

There was no statistically significant difference between the experimental group and control group for PA. Between-group differences in LBP disability (MD= 19.4 units, 95% CI: 8.5, 30.3), functional capacity (primary MD= −4.1 units, 95% CI: −6.9, −1.3; average MD= −3.1, 95% CI: −4.9, −1.2) and self-efficacy (MD −11.3 units, 95%CI −20.2, −2.5) favoured the control group with small to moderate effect sizes. There were low levels of overall engagement with the app.

**Conclusion:**

The embedded MI intervention was no more beneficial than physical therapy alone for PA and was associated with poorer LBP disability, function, and self-efficacy. The effectiveness of embedding MI and a smartphone app into usual care for LBP was not supported.

## Highlights

• Embedded motivational interviewing for low back pain was not superior to usual physical therapy care.
• Embedded motivational interviewing is a new skill for physical therapists.
• Understanding the intended purpose of healthcare apps may foster engagement.

## Introduction

Low back pain (LBP) is a common health complaint and a leading cause of disability worldwide.^1^, ^2^, ^3^ Ten to 15% of people with LBP have experienced chronic LBP lasting for longer than 3 months. Chronic LBP is a burdensome condition and is associated with social isolation, early retirement, and prolonged work absenteeism.^4^^,^^5^ Physical activity may reduce the risk of someone developing chronic LBP by improving physical condition, mood, and motivation.^6^ There is moderate certainty evidence from meta-analysis that physical activity improves absenteeism outcomes in people with sub-acute LBP (4–12 weeks), suggesting this phase may be an important time to target interventions.^7^ Moderate to vigorous physical activity reduces long-term disability in people with chronic LBP,^8^ but physical therapists have reported this can be challenging to facilitate.^9^

Motivational interviewing is an evidence-based counselling technique to address ambivalence towards healthy behaviour change, through relational components (the spirit of motivational interviewing) and technical components (referred to as micro-skills).^10^ Collaboration, autonomy, and evocation of ideas from the patient about behaviour change are facilitated through micro-skills, including open-ended questions, affirmations, reflective listening, and summaries.^10^ Adding face-to-face and telephone-based motivational interviewing to usual physical therapy care improves functional capacity in people with acute to sub-acute LBP.^11^^,^^12^ However, dedicated 1:1 consultations require additional funding and this may not be accessible to all patients. Training physical therapists to allocate a portion of usual treatment time to motivational interviewing may be more time efficient, but in isolation is unlikely to be enough to influence sustained changes in physical activity.

Smartphone applications (apps) have been used as a convenient way to deliver behaviour change interventions in healthcare settings.^13^ Apps incorporating the principles of motivational interviewing have increased self-efficacy and physical activity in sedentary adults.^14^ However, while some components of a motivational interviewing intervention for increasing physical activity are conducive to be being delivered by an app (e.g. questions can be phrased to elicit patient change talk), others (e.g. accurately responding to open ended questions) require face-to-face interactions.^15^ An intervention combining an app-based component with an in-person component that does not significantly detract from other physical therapy treatment modalities, may be one way to address this.

The aim of this trial was to evaluate a new motivational interviewing intervention comprising a physical therapist-delivered component and a self-directed patient app, for increasing physical activity in people with sub-acute LBP.

## Methods

The trial protocol has been published.^16^ A mixed-methods, cluster randomised controlled trial was conducted in the physical therapy outpatient departments of 4 public hospitals in Melbourne, Australia. Hospital sites (clusters) were allocated by single block pragmatic randomisation to deliver 6 weeks of usual physical therapy or 6 weeks of the newly designed motivational interviewing intervention (Table 1). Concealed allocation was completed by an independent researcher using a random number generator (www.randomization.com). The study received ethics approval from the Alfred Hospital Ethics Committee (47/15), Eastern Health Human Research and Ethics Committee (E12-2014), La Trobe University Human Ethics Committee (E12-2014) and Monash Health Human Research Ethics Committee (15067X). The study protocol was listed on the Australian New Zealand Clinical Trials Registry before the trial commenced (12615000724572). All participants provided written, informed consent prior to the trial commencing. The trial is reported in accordance with the Consolidated Standards of Reporting Trials (CONSORT) statement for cluster randomised controlled trials^17^^,^^18^ and the Consolidated Criteria for Reporting Qualitative Research (COREQ)^19^

**Table 1:** Description of interventions.

	Usual Care	Usual Care plus Motivational interviewing
Brief name	Physical therapy for sub-acute low back pain.	Motivational interviewing to increase physical activity in people with sub-acute low back pain.
Why	Reduce symptoms and activity limitations.	Reduce symptoms and activity limitations. Build importance and increase physical activity.
What materials	Regular physical therapy treatment modalities.	Motivational interviewing embedded into regular physical therapy sessions. Self-directed motivational interviewing smartphone application for patients.
Who provided	Physical therapists.	Physical therapists who received 8 h of motivational interviewing training over 2 days.
How provided	In person.	In person (physical therapy component). Smartphone app.
Where (setting)	Outpatient physical therapy department.	Outpatient physical therapy department. A time and place convenient to patients (app component).
When/how much (dose)	6 sessions, each 30 min in duration, over 6 weeks (3 h total).	6 sessions, each 30 min in duration, over 6 weeks (3 h total). Motivational interviewing embedded at the discretion of the physical therapist. Patients prompted to use the smartphone app every 1 to 3 days.
Tailoring	Physical therapy treatment tailored to the patient's requirements and progress.	Motivational interviewing tailored to a patient's levels of importance and confidence. Smartphone app content tailored to patient's level of readiness for change.
Fidelity checking measures	Attendance at physical therapy consultations.	Attendance at physical therapy consultations. Physical therapists’ level of proficiency in delivering motivational interviewing assessed by audio-taped real plays with the study coordinators on the Motivational Interviewing Treatment Integrity Scale.

### Participants

Outpatient physical therapists at participating sites were eligible to take part in the study and recruit patients to the study from their caseloads if they met the following criteria: 3–12 weeks of LBP between the inferior border of the 12th rib and the gluteal fold^20^ preceded by 30 days of no/usual pain,^21^ access to an Apple or Android smartphone and competency using apps requiring text input. Patients were excluded if they had medical red flags (signs or symptoms that may indicate serious pathology),^22^ were waitlisted for surgery, did not speak English, or lived greater than 40 km from the hospital site. Patients who scored in the severe range for depression and/or anxiety on the 21-item Depression, Anxiety and Stress Scale (DASS-21) were also excluded from participating^23^ and follow-up referral with a general practitioner or psychologist was initiated.^16^ Depression and anxiety are associated with an increased risk for developing chronic LBP and may have introduced confounding factors in a trial of this size.^24^

### Interventions

All patients attended a 30-min individualised, face-to-face consultation with their physical therapist once a week for 6 consecutive weeks (Table 1). Physical therapy treatments included, but were not limited to, manual therapy, exercise prescription, advice, and education. Physical therapists in the experimental group attended an 8-hour training program over 2 × 4-h sessions. The program, designed and delivered by a motivational interviewing trainer and physical therapist, aimed to teach physical therapists how to embed components of motivational interviewing into their regular consultations. The content of the program was based on similar motivational interviewing training programs for physical therapists.^11^^,^^25^^,^^26^ Between physical therapy consultations, patients in the experimental group were also instructed to use a new motivational interviewing-based app (MiMate) on their smartphone device. The self-directed app contained 10 sequential modules and comprised a series of specific multiple choice and open-ended questions designed to elicit answers that facilitated change talk towards increasing levels of physical activity. Additional components were a diary for recording physical activity and a flare up module that offered education/suggestions for managing exacerbations of pain. Physical therapists could review patient completed app material in preparation for consultations and patients were informed of this. The app was piloted with a convenience sample of users (*n* = 5), and minor amendments were made to improve usability prior to the trial commencing. The intervention has been described in detail elsewhere.^16^

### Outcomes

Patients were assessed at baseline and at the end of the 6-week intervention by a blinded assessor. The outpatient physical therapy departments were open plan, and to maintain blinding, it was necessary for assessments to be conducted at patients’ residences.

### Primary outcome

Physical activity was assessed as the mean number of daily minutes of moderately vigorous physical activity (MVPA), measured using the activPAL 3 tri-axial accelerometer. The device is a valid and reliable measure of MVPA^27^ and was worn continuously for 7 consecutive days on the antero-lateral thigh.^16^ Data were downloaded using proprietary software.^28^ A daily average was calculated by dividing total weekly MVPA minutes by the number of days the device was worn for 10 or more hours.^29^

### Secondary outcomes

The modified Oswestry Disability Index (ODI) is a 10-item self-report questionnaire that assesses LBP disability as a percentage from 0 (no disability) to 100 (severe disability).^30^ Functional capacity was assessed with the Patient Specific Functional Scale (PSFS).^31^ Patients were asked to rate their ability to perform 1 primary and up to 4 secondary self-selected activities on an ordered scale from 0 (unable to perform) to 10 (perform at pre-injury level). Pain self-efficacy was measured using the Pain Self-Efficacy Questionnaire (PSEQ), a 10-item self-report questionnaire to assess a person's confidence in performing a series of tasks, despite pain.^32^ Each task (item) is scored from 0 (not at all confident) to 6 (completely confident) to yield a total score out of 60.^33^

Physical therapists in the experimental group were assessed for proficiency with the Motivational Interviewing Treatment Integrity (MITI, version 4.2.1) code. The tool assesses the degree to which a recorded interaction is consistent with the technical and relational aspects of motivational interviewing.^34^ Each aspect is rated on an ordinal scale from 1 to 5 with higher scores indicating higher levels of motivational interviewing consistent behaviours. After motivational interviewing training, physical therapists participated in a recorded 20-min session with one of the study coordinators (JH and POH). During the interaction, the study coordinators spoke about a personal health-related behaviour they wanted to change. The audio recording was reviewed by an independent researcher who had completed training in administering the MITI, and the process was repeated 6 weeks into patient recruitment. At the end of the 6-week intervention patients completed the Client Evaluation of Motivational Interviewing (CEMI) questionnaire, a 16-item self-report questionnaire.^35^ Items are scored from 1 (never) to 4 (a great deal) to yield a score out of 64. Higher scores indicate a perceived counselling style that is consistent with motivational interviewing.

### Semi-structured interviews

At the end of patient recruitment, a qualitative process analysis was conducted, to investigate patients’ and physical therapists’ experiences with the intervention. Physical therapists and 12 patients (selected at random) from the experimental group participated in a 30-min recorded semi-structured telephone interview with an independent male researcher, who used an interview guide designed by the research team. De-identified interviews were transcribed by a medical transcription service and downloaded into the NVIVO software package for analysis (Version 12.6.1.970, QSR International, Burlington, Massachusetts). Interviewees received a $50 retail voucher as an acknowledgement of their time.

Adverse events were recorded and followed up according to the policies of the treating healthcare site.

### Analysis

To achieve 80% power at a 0.05 significance level assuming a large effect size for the primary outcome and an intraclass cluster coefficient of 0.05, 14 participants per cluster were required.^36^^,^^37^ The Final recruitment target was 15 per cluster (60 in total), allowing for loss to follow-up. Intention to treat principles were applied to all analyses.^38^ Further details regarding the sample size calculation can be found in the trial protocol.^16^

Between-group differences at the end of the 6-week intervention for the primary and secondary outcomes were tested with analysis of covariance (ANCOVA), entering mean group and cluster baseline scores as covariates.^39^ Age and symptom duration (at the time of the first physical therapy consultation in the study) are known predictors of LBP chronicity and were entered as additional co-variates.^40^ Between-group differences in physical therapy attendance and accelerometer wear days were analysed by independent *t*-tests. Changes in motivational interviewing proficiency across time were assessed by paired *t*-tests.

### Qualitative analysis

Interview transcripts were analysed inductively by interpretive description.^41^ A random selection of 4 patient and 2 physical therapist transcripts were reviewed by two authors for common excerpts of interest, grouped together to form sub-themes. This process continued until no additional patterns were identified (data saturation). Common physical therapist and patient sub-themes were combined under a series of major themes and presented narratively.

## Results

**Fig. 1:**
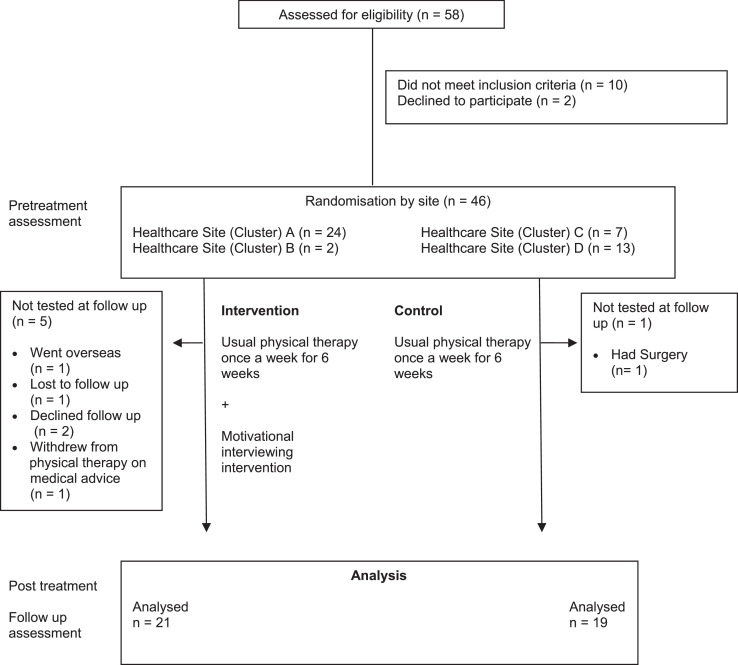
Trial design and flow of participants through the study.

Patient recruitment commenced on 27 March 2017, and the final assessment was completed on 23 August 2018. The trial was finalised before meeting the recruitment target because of resourcing constraints. The flow of patient participants through the study is shown in Fig. 1. Of 58 patients screened for eligibility, 2 declined because their symptoms improved, 10 were excluded because they scored in the severe range for depression and/or anxiety on the DASS-21, and 46 were enrolled (20 in the control group and 26 in the experimental group). One patient in the control group and 5 patients in the experimental group did not complete a follow-up assessment. Two patients in the experimental group experienced a mild skin reaction to the accelerometer film and did not complete this component of the reassessment and 1 patient was unavailable for the follow-up accelerometer assessment. These data were omitted from the follow-up assessment of the primary outcome. All available data were included in the analysis.

There were 78 physical therapy consultations in the control group (mean = 4.6, standard deviation [SD] 1.6) and 72 consultations in the experimental group (mean = 3.2, SD 1.80), a mean difference of 1.3 consultations (95% CI: 0.3, 2.4). Nine participants in the experimental group (35%) did not use the MiMate app at all. The average number of core modules completed over the 6-week intervention was 4.8 (SD 3.9). The average number of activity diary entries was 25.4 (SD 34.7) and the flare up module was used an average of 2.0 (SD 2.9) times.

The mean age of participants was 43.7 (SD 14.3), and the mean symptom duration at the time of the first treatment session was 30.2 (SD 14.6) days (Table 2 and supplementary material).

**Table 2:** Baseline characteristics of patient participants by group.

Demographics	Exp *n* = 26	Con *n* = 20
Age (years)	39.2 (12.8)	49.5 (14.9)
Sex Male Female	8 (31%) 14 (69%)	10 (50%) 10 (50%)
Symptom duration* Baseline assessment First treatment session	28.0 (16.1) 35.2 (16.5)	23 (8.7) 23.6 (8.1)
DASS-21 Anxiety subscale (0–42)…) Depression Subscale (0–42))	4.2 (4.1) 5.2 (5.2)	4.6 (4.0) 6.1 (6.3)
Employment status Full time hours Part time hours Casual hours No paid employment Missing	10 (39%) 5 (19%) 1 (4%) 7 (27%) 3 (12%)	11 (55%) 3 (15%) 0 (0 %)6 (30 %) 0 (0 %)
Receiving sick leave entitlements Yes No N/A Missing	6 (23%) 12 (46%) 7 (27%) 1 (4%)	4 (20%) 6 (30%) 7 (35%) 3 (15%)

Data are mean (standard deviation), frequency (proportion).

Abbreviations: Exp, experimental group; Con, control group; DASS, Depression.

Anxiety and Stress Scale.

*Days since the onset of low back pain.

### Effect of the intervention

At the end of the 6-week intervention there was no statistically significant difference between the control group and experimental group for the primary outcome measure, mean daily minutes of MVPA (mean difference= 0.9 min, 95% CI: −6.7, 8.6) (Table 3).

**Table 3:** Mean ± standard deviation within groups and mean difference (95% CI) difference between groups at the end of the 6-week intervention for the primary and secondary outcomes.

	Usual Care (*n* = 26)	Usual Care plus Motivational Interviewing (*n* = 20)	Between-group difference in change scores
	Mean ± SD	Mean ± SD	Mean (95% CI)
MVPA^a^			
Pretreatment	9.7 (9.6)	8.1 (10.6)	
Posttreatment	11.7 (16.1)	9.7 (9.6)	1.0 (−6.6, 8.6)
Oswestry Disability Index (0–100) b			
Pretreatment	34.8 (18.3)	38.1 (15.2)	
Posttreatment	15.8 (14.0)	26.7 (16.9)	19.4 (8.5, 30.3)⁎⁎
PSFS: Primary Item (0–10)			
Pretreatment	3.1 (1.9)	3.7 (2.4)	
Posttreatment	8.0 (2.7)	5.6 (2.3)	−4.1 (−6.9, −1.35)⁎⁎
PSFS: Item Average (0–10)			
Pretreatment	3.7 (1.2)	3.4 (1.5)	
Posttreatment	8.0 (2.1)	5.3 (1.9)	−3.1 (−4.9, −1.2)⁎⁎
PSEQ (0–60)			
Pretreatment	38.3 (13.9)	34.1 (10.9)	
Posttreatment	51.1 (8.7)	41.8 (13.0)	−11.3 (−20.2, −2.5)*

Abbreviations: MVPA, Moderate to Vigorous Physical Activity; PSFS, Patient Specific Functional Scale; PSEQ, Pain Self Efficacy Questionnaire.

a Average daily minutes.

b Lower score signifies better function iAdjusted scores from ANCOVA using group and cluster baseline scores, age and symptom duration at first consultation as covariates.

⁎ <0.05.

⁎⁎ *p* < 0.01.

Between-group differences in pain disability, function, and self-efficacy favoured the control group at the end of the 6-week intervention. The mean differences were 19.4 units for the ODI score (95% CI: 8.5, 30.3), 4.1 units for the PSFS primary item (95% CI: 1.3, 6.9), 3.1 units for the PSFS item average (95% CI: 1.2, 4.9), and 11.3 units for the PSEQ (95% CI: 2.5, 20.22).

### Therapist proficiency

All 5 physical therapists in the experimental group were proficient in motivational interviewing after training. The mean score for the MITI was 3.4 (SD 0.2) for the technical subscale and 3.8 (SD 0.3) for the relational subscale. There was no significant change in either MITI sub-scale score at follow up assessment (technical sub-scale 3.4 (SD 0.2); relational sub-scale 3.7 (SD 0.5). The mean score on the CEMI was 50.7 (SD 6.1) (*n* = 20).

### Adherence to trial protocol

In a variation from the trial protocol^16^ 26 of the 83 assessments were completed by a study coordinator (JH) due to resourcing challenges. These assessments were therefore unblinded.

### Qualitative findings

Twelve of 15 patients completed an interview. Four of the 5 physical therapists in the experimental group were interviewed. Three major themes were identified from 7 therapist and 7 patient sub-themes (Table 4).

**Table 4:** Qualitative Findings: Major themes, sub-themes, and extracts from the semi structured interviews.

Major Theme		Participant sub-theme	Example interview extract	Physical therapist sub-themes	Example interview extract
Therapeutic style		Patient/therapist connection	“They just got me.” (Pt 07) “They (treating physical therapists) never made (me) feel like just a patient” (Pt 12)	Motivational interviewing is a different mindset that requires a different skillset	“Reflections were something new, so sort of making sure obviously you're listening to what they're saying and then almost repeating it back to them to show that – “Yeah, I hear what you said. (Tx02)
		Physical therapy consultations were different to previous experiences with healthcare providers	“I felt more taken care of by the staff and they explained things a lot more. They asked more about my lifestyle and how my back affected my everyday living and what I wanted out of the physio” (Pt05)	Motivational interviewing helps build rapport with patients	“You definitely build a lot more rapport with patients using motivational interviewing. They're maybe more open to what you're saying. A lot of patients maybe just feel they're being told what to do all the time, as opposed to being listened to.” (Tx03)
Therapeutic content and implementation		Setting goals as a team	“They would always ask me how I think I would go-not just give me an activity and say – you've got to do this physical activity… It wasn't just giving me things that I had to do; we'd have a discussion about it.” (Pt 11)	Emphasis on collaborative goal setting	“It was useful to try and find out where they were at in terms of how ready they were to change or increase (their activity levels)” (Tx 02)
		Physical therapists were good communicators who wanted to understand	“They were very interested, engaged, and they wanted me to get better and were there to support that” (Pt10)	Embedding motivational interviewing was a helpful assessment tool	“They were just relaxed and chatting, but I was getting useful information and getting them to think about what they wanted to achieve and how confident they felt they were, without them really thinking I was questioning them.” (Tx02)
		Low levels of engagement with and use of the MiMate app	“It was just time consuming; we all have busy schedules.” (Pt 10) “I remember (the physical therapist) saying that she was on the other side of the app. But I don't really feel like I got a lot back from that.” (Pt03)	Checked app compliance initially, stopped asking if the patient seemed disinterested	“If consistently over a few sessions they weren't using it, and they didn't show interest in it, I stop asking them.” (Tx 04)
Impact and suggested improvements		Examples about how to use the MiMate app and how it may be used as a part of physical therapy treatment	“There just needed to be a bit more current explanation of what the app actually did. A video case study of how to use it would definitely be a good thing” (Pt07)	Training program could have been more physical therapy-specific	“It might have been helpful to see a physical therapist implementing it exactly how we would.” (Tx 02)
		Ongoing impact	“The treatment) helped me. Now, I'm regular with my exercise and that's helped me to get back and get rid of my back pain which is a very important thing for me” (Pt08)	Ongoing use of motivational interviewing	“I found it was really helpful to implement, not just with the patients that were involved with the trial, but also any of the outpatients that I was seeing.” (Tx02) ” I think definitely the theme of motivational interviewing has been really good and has changed the way I approach patients” (Tx03)

Abbreviations: Pt, Patient; Tx, Physical therapist.

Major theme 1, therapeutic style: All physical therapists described motivational interviewing as a different way of communicating, requiring them to speak less and listen more. Three physical therapists felt using motivational interviewing helped them build greater rapport with their patients. This was mirrored in comments 11 patients made about feeling a strong sense of connection with their physical therapist.

Major theme 2, therapeutic content and implementation: Seven patients discussed working collaboratively to set activity related goals. This was consistent with how 3 physical therapists described using reflections and summaries to facilitate collaborative goal setting.

Four patients reported using the MiMate app regularly over the 6-week intervention, 6 intermittently and 2 did not use it at all. Barriers to app use included uncertainty of purpose, lack of perceived benefit over required effort, and ambiguity regarding some open-ended questions. Three physical therapists reported asking about the app initially, but stopped if they perceived patients were not interested.

Major theme 3, impact/suggested improvements: Three physical therapists felt the training could have been more specific to outpatient physical therapy environments, through video examples of physical therapists embedding motivational interviewing. Three patients also suggested videos may be an efficient way to introduce the app.

## Discussion

This trial investigated a new way of delivering motivational interviewing that combined a face-to-face component (physical therapy embedded motivational interviewing) and a self-directed patient smartphone app, MiMate. There was no between-group difference in change in physical activity at the end of the 6-week intervention. The recruitment target of 60 participants was not met and this likely contributed to the study being underpowered for the primary outcome measure (MVPA).

Improvements in LBP disability, functional capacity, and pain self-efficacy favoured the control group, with small to moderate associated effect sizes. These findings are in contrast with a previous study that showed 6 × 30-min telephone motivational interviewing consultations plus physical therapy improved functional activity in patients with sub-acute LBP, compared with physical therapy alone.^11^ In the current study motivational interviewing was embedded into usual physical therapy consultations and there were poor levels of compliance with the patient smartphone app. The amount of motivational interviewing delivered to patients is likely to have been less than in previous studies.^11^^,^^12^^,^^42^ In the current trial, patients in the control group also received more physical therapy (on average 4.6 vs 3.2 consultations). This can make findings difficult to interpret. Therefore, patients in the experimental group received less physical therapy care, and the intensity of motivational interviewing delivered may not have been sufficient to influence a meaningful increase in MVPA. A previous systematic review and meta-analysis found that brief sessions of motivational interviewing of 15 or more minutes were potentially effective for facilitating health behaviour change in people with chronic health conditions.^43^ However, the motivational interviewing interventions included for review were either dedicated face-to-face or group interactions. The findings of the current trial support the need for further randomised controlled trials to evaluate how to best integrate motivational interviewing into regular healthcare practice in non-counselling settings.

This study had several strengths. Physical therapists’ proficiency in delivering motivational interviewing was confirmed using a validated outcome measure. The cluster design also meant that there was a small risk of contamination between experimental and control physical therapists. Despite not meeting the recruitment target, the study was likely sufficiently powered to detect a statistically significant difference for the secondary outcome measures.

There were also some limitations. The recruitment target of 60 participants was not met and one healthcare site only recruited two patient participants, because of an unexpected decline in patient referrals for LBP. There were low levels of engagement with and use of the MiMate, which was designed to increase the amount of motivational interviewing delivered to patients. The MiMate smartphone app and online therapist portal were delivered as intended (patients were able to download it and functionality of the therapist portal was confirmed). However, it appears likely that these components of the intervention were not used as intended by most patients and physical therapists. The main app modules were designed to be accessed every 1–3 days; however, patients used this section on average only 4.8 times over the 6-week intervention and 35% did not use it at all. None of the physical therapists reported using the therapist portal to review patient completed app content. The online portal was envisaged as the conduit between the two components of the intervention and designed to assist physical therapists in planning the motivational interviewing content of consultations. Future studies may consider using motivational interviewing as part of the physical therapy training program to improve patient compliance with the app, as well as therapists' use of the online portal. Some patients reported they were unsure about the relationship between the app and physical therapy consultations. Uncertainty of purpose is a barrier to patients engaging with digital interventions for LBP.^44^ A series of introductory videos within the MiMate app that explain its purpose and provide examples of it being used in everyday situations may be a practical way of addressing this.

Finally, physical therapists were assessed for proficiency in delivering motivational interviewing by a 20-min face to face session with a study coordinator, who spoke about a personal health-related behaviour they wanted to change. This was not aligned with how physical therapists were taught to embed motivational interviewing into regular consultations for LBP and the extent to which physical therapists were able to achieve this remains uncertain. Audio-recording all physical therapy consultations and applying the MITI to a random selection of de-identified consultations would provide a more accurate assessment.^45^

## Conclusions

It remains uncertain if training physical therapists to embed motivational interviewing into consultations, and combining this with a self-directed patient app, is more beneficial than usual physical therapy care for increasing physical activity in sub-acute LBP. Despite this, physical therapists were positive about motivational interviewing. Given the accessibility and potential cost-effectiveness of evidence-based behaviour change apps, further studies are warranted to establish the feasibility and effectiveness of the intervention. These should ensure adequate steps to optimise patient adherence and engagement with the MiMate smartphone app.

## Conflicts of Interest

The author declares no conflicts of interest.

## Appendix Supplementary materials

Image, application 1
