# Supplementary material for: Embedded motivational interviewing combined with a smartphone application to increase physical activity in people with sub-acute low back pain: a cluster randomised controlled trial
Source: Braz J Phys Ther. 2024 Jun 16;28(4):101091. doi: 10.1016/j.bjpt.2024.101091 (PMC11260563; doi:10.1016/j.bjpt.2024.101091)
Supplement: Supplementary file 1 [file mmc1.pdf]

Supplementary material online

Baseline demographics by cluster

	Exp<br>Cluster A<br>n = 24	Cluster B<br>n = 2	Con<br>Cluster C<br>n = 7	Cluster D<br>n = 13
Age (years)	38.8 (12.7)	44.0 (5.7)	44.3 (8.6)	52.4 (12.9)
Sex				
Male	8 (33%)	0 (0%)	5 (71%)	5 (38%)
Female	16 (67%)	2 (100%)	2 (29%)	8 (62%)
Symptom duration†				
Baseline assessment	28.8 (16.2)	19.5 (16.3)	25.9 (9.4)	21.5 (8.3)
First consultation	36.1 (16.7)	25.0 ± 11.3	23.7 ± 11.1	23.5 (6.4)
DASS-21				
Anxiety subscale	4.6 (4.1)	0 (0)	2.9 (2.8)	5.5 (4.3)
Depression subscale	5.6 (5.3)	1.0 (1.4)	4.9 (4.7)	6.8 (7.1)

Data are mean ± standard deviation, frequency (proportion)

Abbreviations: Exp, experimental group; Con, control group; DASS, Depression, Anxiety and Stress Scale

†Days since the onset of low back pain
